# A novel method for assessing the development of speech motor function in toddlers with autism spectrum disorders

**DOI:** 10.3389/fnint.2013.00017

**Published:** 2013-03-28

**Authors:** Katherine Sullivan, Megha Sharda, Jessica Greenson, Geraldine Dawson, Nandini C. Singh

**Affiliations:** ^1^Department of Psychology, Center on Human Development and Disability, University of Washington Autism CenterSeattle, WA, USA; ^2^Department of Cognitive Neuroscience and Neuroimaging, National Brain Research CentreGurgaon, India; ^3^Department of Psychiatry, University of North Carolina at Chapel HillNC, USA

**Keywords:** autism, language, vocalizations, speech motor development, articulatory features

## Abstract

There is increasing evidence to show that indicators other than socio-cognitive abilities might predict communicative function in Autism Spectrum Disorders (ASD). A potential area of research is the development of speech motor function in toddlers. Utilizing a novel measure called “articulatory features,” we assess the abilities of toddlers to produce sounds at different timescales as a metric of their speech motor skills. In the current study, we examined (1) whether speech motor function differed between toddlers with ASD, developmental delay (DD), and typical development (TD); and (2) whether differences in speech motor function are correlated with standard measures of language in toddlers with ASD. Our results revealed significant differences between a subgroup of the ASD population with poor verbal skills, and the other groups, for the articulatory features associated with the shortest-timescale, namely place of articulation (POA), (*p* < 0.05). We also found significant correlations between articulatory features and language and motor ability as assessed by the Mullen and the Vineland scales for the ASD group. Our findings suggest that articulatory features may be an additional measure of speech motor function that could potentially be useful as an early risk indicator of ASD.

## Introduction

Autism spectrum disorder (ASD) is an early onset, complex, and pervasive developmental disorder characterized by significant impairments in social and communication development as well as repetitive and restricted behaviors and interests. Impairments in communication associated with ASD include delayed onset of babbling (Iverson and Wozniak, 2007), unusual or absent communicative gestures (Baranek, 1999; Mitchell et al., 2006), diminished responsiveness (Mitchell et al., 2006), lack of non-verbal and verbal integration (Tager-Flusberg et al., 2005), aberrant patterns of sound production (Wetherby et al., 1989), and odd vocal quality (Sheinkopf et al., 2000). While some children remain non-verbal, these numbers appear to be dropping with advances in early identification and implementation of early intervention (Tager-Flusberg et al., 2005).

Young children with ASD, who begin to use and experiment with speech, produce babbles and vocalizations that are often unusual in tone and include repetitive screeching, groaning, humming, or echolalia (Tager-Flusberg and Caronna, 2007). A common focus of previous studies examining speech production in children with ASD was to identify such patterns of atypicality in their vocalizations. For instance, reports of slow and unusual speech patterns were described as one of the earliest symptoms of ASD (Lord and Paul, 1997). Findings from prospective and retrospective studies using videotapes of toddlers demonstrate differences in linguistic abilities including communicative intent and use of spoken language in children with ASD as early as 2 years of age (Dahlgren and Gillberg, 1989; Sheinkopf et al., 2000; Landa and Garret-Mayer, 2006). Studies on vocal atypicalities in children with ASD have focused on describing the aberrant nature of phonological output in terms of proportion of syllables with atypical phonation as well as odd vocal quality (Sheinkopf et al., 2000). Other reports have shown that the rate of acquiring language in ASD is often slower than other children who have language delays, which may be related to level of cognitive functioning, whereas for other children it may lag behind development in other areas (Lord and Pickles, 1996).

In recent years, a number of research studies have used early vocalization data to examine and characterize differences in children with ASD compared to typically developing children (Cleland et al., 2010; Oller et al., 2010; Schoen et al., 2011; Shriberg et al., 2011). However, most of these studies have done so in the context of social functions and reciprocity. Deficits in the development of speech and language function in this population have been associated with impairments in orienting to social stimuli such as faces as well as poor performance on joint attention tasks (Bernabei et al., 1998; Mars et al., 1998; Baranek, 1999; Osterling et al., 2002). However, there is now increasing evidence to indicate that a lack of communicative intent in the form of speech or gestures in children with ASD may be related to issues other than social-cognitive abilities (Prizant, 1996). A potential area for such investigation is general motor or more specifically speech motor function. In this context, speech production tasks may provide a useful way to examine oral-motor skills associated with speech motor function and vocalization patterns in individuals with ASD. Recent work has shown that early childhood measures of oral-motor and manual motor skills can predict later speech fluency in children with ASD (Gernsbacher et al., 2008), and may be better predictors of later speech abilities than measures of social cognition (Thurm et al., 2007).

In the current study, we explored motor aspects of speech production to better understand and characterize the vocalization deficit in children with ASD. We sought to determine whether differences in speech motor function are found in young children with ASD as compared to age-matched children with typical development (TD) and developmental delay (DD), and if so, whether such differences are associated with individual variation in spoken language ability. We employed a quantitative measure of speech motor function, referred to as “articulatory features,” to identify such discrepancies in vocalizations and in the development of speech motor control. This measure is based on acoustic differences in vocalization patterns and assesses articulatory features derived from spectrotemporal analysis of a collected speech sample. Vocal learning critically depends on the ability to perceive and categorize sounds at different timescales (Doupe and Kuhl, 1999). For example, the amplitude envelopes for vowels fluctuate at a long-timescale of hundreds of milliseconds while those for consonants fluctuate at a shorter-timescale of tens of milliseconds (Rosen, 1992). Given that past research has shown that children with ASD show atypical temporal processing, we hypothesized that that such atypicality may possibly be captured in the timescale characteristics of speech production. In the current study, we employed a quantitative measure of speech motor function and suggest that vocal production patterns may be classified into “articulatory features” of two kinds, those involving slower amplitude fluctuations (vowel-like, at hundreds of milliseconds) and those involving faster amplitude fluctuations (consonant-like, at tens of milliseconds).

Previous research demonstrates a specific developmental time course of these articulatory features in typically developing children, and has been shown to reflect the maturation of speech motor control (Singh et al., 2007; Singh and Singh, 2008). Initially, children develop fine articulatory-motor maps wherein they learn to organize these articulatory features to produce fluent speech. This occurs between middle to late childhood, possibly during the process of sensori-motor integration. In addition, these features can be used as a metric to examine the nature of consonants, vowels, blends, and transitions used by the toddlers while their oromotor apparatus is still developing. As mentioned above, research involving speech features is relatively new and has not been established as a standard measure among individuals with ASD. Research is expanding in this area, however, and new developments in automated technology for vocal analysis of toddlers with ASD (Oller et al., 2010) may lead to the use of vocalizations as an early risk indicator for ASD and the general study of language development.

Additionally, an important focus of future research will be to assess how well-speech features correlate with well-established measures of communication and language, such as parent reports/questionnaires [e.g., the Vineland Adaptive Behavior Scales (Venter et al., 1992; Toth et al., 2006; Sutera et al., 2007; Thurm et al., 2007), the Autism Diagnostic Interview-Revised (ADI-R; Sutera et al., 2007; Thurm et al., 2007), the Sequenced Inventory of Communication Development (Thurm et al., 2007), and the MacArthur-Bates Communicative Development Inventory: the Words and Sentences/Words and Gestures (Smith et al., 2007)] and behavioral observations [e.g., the Autism Diagnostic Observation Schedule-Generic (ADOS; Sutera et al., 2007; Thurm et al., 2007), the Mullen Scales of Early Learning subscales (Venter et al., 1992; Toth et al., 2006; Smith et al., 2007; Sutera et al., 2007; Thurm et al., 2007), and the Differential Ability Scales (Sutera et al., 2007)].

In summary, in the current study, methods of spectral analysis were used to assess articulatory features of a collected speech sample from children with ASD, DD, and TD. We sought to expand previous research on use of articulatory features to assess speech motor function in two ways: (1) by examining these features in a sample of toddlers with ASD as compared to toddlers with developmental delay (DD) and typically developing (TD) toddlers; and (2) by evaluating the relationship between articulatory features and well-established measures of communication and language among young children with ASD. These measures include the Mullen Scales of Early Learning (Mullen, 1997) and the Vineland Adaptive Behavior Scales (Sparrow et al., 1984). If differences in speech production are identified between young children with ASD, TD, and DD in the current study, articulatory features may be indicated as a measure for identifying early risk for ASD as well as a predictor of developmental trajectories of language in this population.

## Material and methods

### Participants

Participants were recruited as part of the National Institute of Mental Health (NIMH)-funded University of Washington (UW) Early Studies to Advance Autism Research and Treatment (STAART) study. The sample consisted of three groups: (1) 39 toddlers with ASD, (2) 26 chronological age-matched typically developing children, and (3) 20 chronological and mental age-matched children with idiopathic DD (see Table 1 for detailed demographic information). The DD group was matched to the ASD group on a measure of non-verbal mental age. This variable was computed from averaging age-equivalent scores on the Mullen Scales of Early Learning visual reception and fine motor scales (Mullen, 1997). The Mullen is a standardized measure used to assess the developmental level of children from birth to 68 months. As mentioned above, the DD group was also matched to the ASD group on chronological age. Participants were recruited from pediatric practices, birth-to-three centers, preschools, hospitals, and state and local autism organizations. The ethnicities of participants reflect the minority distribution of the wider Seattle area. Male to female ratio for the ASD group is ~3:1 (Males, *n* = 29; Females, *n* = 10). Data for the current study were collected at baseline of the STAART study before any experimental intervention began. Any private and community-based interventions that ASD participants were receiving outside of the STAART study were documented using an intervention history interview. Exclusionary criteria included a neurological disorder of known etiology (e.g., Fragile X), significant sensory or motor impairment, major physical abnormalities, history of serious head injury, and/or neurological disease.

**Table 1:** **Clinical characteristics and behavioral measures for ASD, TD, and DD groups**.

	**ASD group *n*** = 39		**TD group *n*** = 26		**DD group *n*** = 20		***F***	***p***
	**Mean (*SD*)**	**Range**	**Mean (*SD*)**	**Range**	**Mean (*SD*)**	**Range**		
Age at study entry, mos	23.5 (3.8)	18–30	23.1 (3.0)	18–29	22.1 (3.5)	18–30	1.01	0.368
**GENDER**								
Male (%)	29 (74)	–	19 (73)	–	17 (85)	–	χ^2^(2) = 1.07	0.585
Female (%)	10 (26)	–	7 (27)	–	3 (15)	–		
**MULLEN**								
Early-learning composite^a^	59.4 (16.0)	24–95	105.2 (7.7)	94–127	79.1 (10.7)	57–108	100.77	<0.001
Mullen receptive language^b^	22.2 (7.2)	20–56	57.4 (6.8)	40–78	37.2 (13.3)	20–69	123.59	<0.001
Mullen expressive language^b^	26.9 (9.2)	20–56	48.1 (8.7)	30–68	32.5 (7.6)	20–46	47.36	<0.001
Mullen fine motor^b^	32.1 (11.6)	20–50	49.8 (6.4)	39–64	35.7 (12.8)	20–66	23.63	<0.001
**VABS**								
Adaptive behavior composite^a^	69.2 (6.9)	57–86	95.2 (8.3)	81–115	78.5 (8.9)	64–97	85.21	<0.001
Receptive language^c^	11.1 (3.4)	5–28	14.6 (0.9)	13–16	13.3 (1.3)	10–15	17.37	<0.001
Expressive language^c^	5.8 (2.3)	2–12	11.6 (1.8)	8–15	8.1 (1.4)	6–11	69.51	<0.001
**ADOS**								
Severity score	7.3 (1.7)	4–10	1.6 (1.0)	1–4	2.2 (1.9)	1–9	125.50	<0.001
Social total	11.6 (2.3)	6–14	1.5 (1.4)	0–5	4.0 (3.1)	0–13	168.49	<0.001
Communication total	5.5 (1.6)	2–9	1.1 (1.0)	0–3	2.0 (2.0)	0–8	73.76	<0.001
Repetitive total	2.7 (1.6)	0–6	0.5 (0.7)	0–2	1.1 (1.4)	0–4	23.28	<0.001
**ADI-R**								
Social score	16.4 (3.7)	9–25	–	–	6.3 (3.4)	1–12	51.46	<0.001
Communication score	11.7 (1.8)	6–14	–	–	5.3 (3.3)	0–12	52.13	<0.001
Repetitive score	3.6 (2.0)	0–8	–	–	1.6 (1.1)	0–4	8.40	<0.001

Notes: ASD, autism spectrum disorder; TD, typically developing; DD, developmentally delayed; VABS, Vineland Adaptive Behavior Scales; ADOS, Autism Diagnostic Observation Scale; ADI, Autism Diagnostic Interview—Revised.

a *Standard score (mean:100 [SD:15])*.

b *T score (mean: 50 [SD:10])*.

c *VABS Subdomain V-score (mean: 15 [SD:3])*.

All participants were administered the ADOS (Lord et al., 1989, 1999). ASD and DD participants' parents were also administered the ADI-R (Lord et al., 1994) for diagnostic clarification (i.e., developmental delays vs. developmental deviances characteristic of ASD). Given that TD participants did not meet diagnostic criteria for ASD on the ADOS or show elevated symptoms, their parents were not administered the ADI-R. In addition to these instruments, study clinicians made a clinical judgment of diagnosis based on presence or absence of symptoms of ASD as defined in the DSM-IV (American Psychiatric Association, 2000). If a child received a diagnosis of autism based on the ADOS and clinical diagnosis, and came within two points of meeting criteria on the ADI-R, the child was considered to have an ASD. In addition, participants from all three groups were administered the Mullen Scales of Early Learning and the Vineland Adaptive Behavior Scales: Expressive and Receptive language subdomains (see Table 1 for detailed scores).

### Methods

#### Speech samples

In order to capture an accurate representation of each toddler's naturalistic speech, two contexts were used for speech sampling: (1) the ADOS and (2) a parent-child interaction (PCI) measure developed by the UW Autism research team. Both the ADOS free play activity and the PCI measures were video- and audio-taped by trained research assistants for later analysis.

The ADOS (Lord et al., 1999) is a semi-structured, interactive schedule designed to assess social and communicative functioning among those who may have ASD. The assessment involves the presentation of a variety of social occasions and “presses” designed to elicit behaviors relevant to diagnosing ASD. The schedule consists of four developmentally sequenced modules of which only one is administered, depending on the examinee's expressive language ability. Due to the age and language ability of the participants in the current study, all children were evaluated using either Module 1 or 2. ADOS Modules were administered by advanced graduate students or licensed psychologists who had achieved reliability on these ADOS Modules. One item included in the ADOS is called “Free Play,” during which toddlers were presented with an assortment of objects and toys. Both the examiner and a parent were in the room, however, the parent was asked to simply observe and respond only if their child initiated contact. Approximately halfway through the free-play activity, the examiner attempted to interact with the child. Length of the free play activity varied for each participant. Any speech uttered by the toddlers during the free play activity was included in that participant's speech sample.

During the PCI measure, the children interacted with one of their parents (almost always the mother) for 6 min in an examination room. The children and their parents were provided with a standard set of toys and participants were asked to play and interact with each other as they would at home. Any speech uttered by the toddlers during the PCI was included in that participant's speech sample.

Speech samples from the ADOS and PCI were combined to form one audio for each participant. All audio files were 16-bit digitized and sampled at a rate of 22 kHz. A trained researcher edited out any adult tokens or environmental sounds within these samples. The file obtained included 2–5 min of naturalistic speech samples for each child that was used to extract a measure of the child's “articulatory features.”

#### Articulatory features

**Figure 1:**
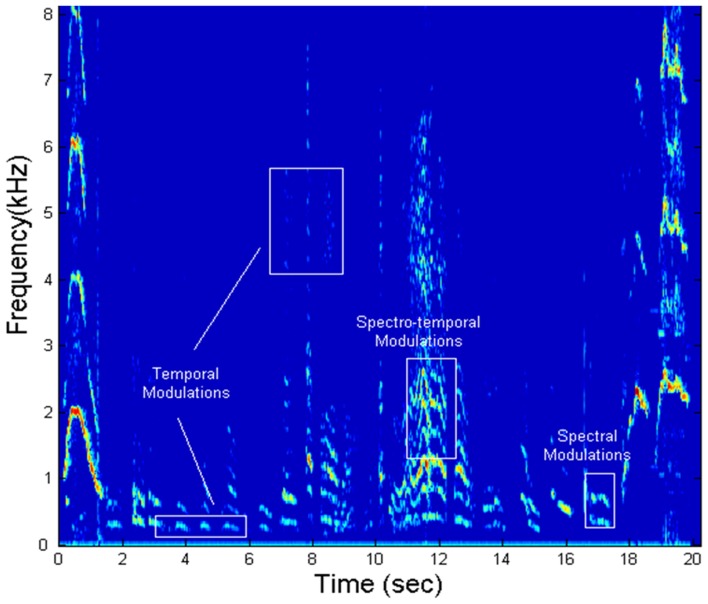
**Representative spectrogram of vocalizations in a toddler's speech sample, demonstrating spectro-temporal modulations**.

Speech is a signal that involves processing at multiple timescales (Rosen, 1992). It is therefore proposed that articulatory features of spoken language require the sensori-motor integration of articulatory gestures at different timescales. Singh and Singh (2008) developed a novel spectral analysis technique, called Speech Modulation Spectrum to study the organization of such articulatory gestures as a metric of speech motor skills. The first step of this analysis involves using speech samples from each participant to calculate a spectrogram. The spectrogram is a time-frequency representation of the speech signal and offers a visual display of fluctuations in frequency and time (see Figure 1), described respectively as spectral and temporal modulations. As shown in Figure 1, spectral modulations (ω_f_) are energy fluctuations across a frequency spectrum at particular times, whereas temporal modulations (ω_t_) are energy fluctuations at a particular frequency over time. Based on the rate of fluctuation, spectro-temporal modulations have been proposed to encode three articulatory features, namely (1) syllabicity or syllabic rhythm (SR) (2–10 Hz), (2) formant transitions (FT) reflecting consonant blends and transitions (20–40 Hz), and (3) place of articulation (POA) reflecting finer, rapid-scale changes in utterance (50–100 Hz).

**Figure 2:**
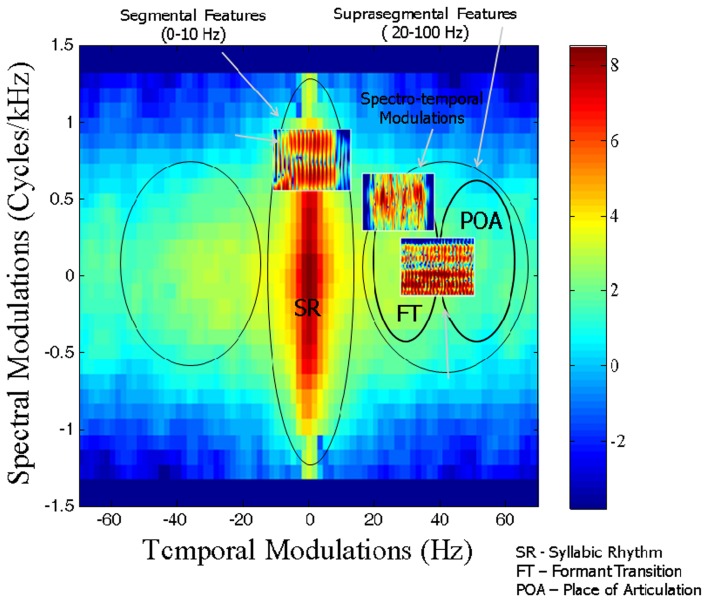
**Representative Modulation Spectrum derived from Spectrogram in Figure 1 by carrying out a 2-D Fourier decomposition, demonstrating the presence of articulatory features as a function of spectro-temporal modulations**.

**Figure 3:**
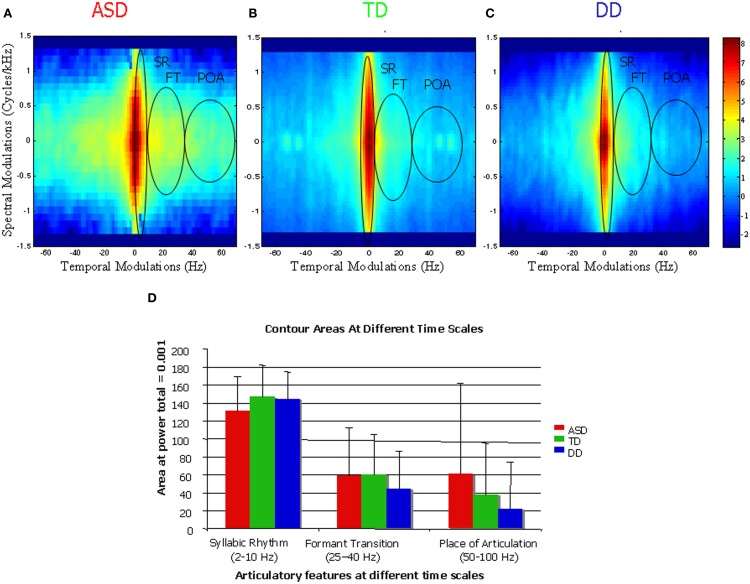
**Contour areas encompassing 99.9% energy in the distribution of spectro-temporal modulations in representative speech samples of the three groups—ASD (A), TD (B), and DD (C).** Panel **(D)** shows a quantification of the energy in the three features across all three groups. Notice higher energy at shorter time scales (FT and POA) indicated in yellow for ASD children as compared to TD and DD groups also quantified in **(D)**.

A 2-D Fourier transform of the spectrogram yields a probability distribution of these different articulatory features and is called the Speech Modulation Spectrum (Singh and Theunissen, 2003). In a typical speech modulation spectrum, the central region between 2 and 10 Hz carries supra-segmental information and encodes SR. The side lobes between 10 and 100 Hz carry information about segmental features. FTs are encoded between 25 and 40 Hz, and POA information is found between 50 and 100 Hz (Stevens, 1980; Tallal et al., 1985). As the modulation spectrum goes from 1 to 100 Hz, the amplitude fluctuations of a sound become faster and go from syllabic to vowel-like to plosive-like segments (Singh et al., 2007). The modulation spectrum thus plots a “language articulation map,” which depicts how energy or “power” is distributed in different articulatory features of spoken language, namely SR, FT, and POA (see Figure 2). Quantifiers to investigate speech features included contour areas at the three different timescales of SR, FT, and POA. The contour area defined in Figure 3 is the total number of spectro-temporal modulations that encompass 99.9% of the total energy. The total contour area, therefore, is comprised of the number of spectro-temporal modulations for each articulatory feature. The contour area for each articulatory feature is the number of modulations as defined by the temporal limit for that feature—thus the contour area for SR is the number of spectro-temporal modulations between 0 and 10 Hz, for FT the spectro-temporal modulations between 10 and 50 Hz and for place for articulation between 50 and 100 Hz. Speech Modulation Spectra for the current study were created from samples that were analyzed for articulatory features by trained raters unaware of each child's diagnosis. For more details on the method please refer to Singh and Singh (2008).

In the same study by Singh and Singh (2008), Speech Modulation Spectrum analysis performed on speech samples of 160 typically developing children 4–8 years old demonstrated a developmental pattern for the three articulatory features described above: (1) adult-like patterns of syllabicity (2–10 Hz) emerged at 4 years old or earlier, (2) FT emerged by 5 years old, and (3) POA emerged by 6–7 years old and beyond (Singh and Singh, 2008). These results demonstrate that in the typical course of development, children exhibit increasingly more power in features associated with shorter-times scales (i.e., POA), possibly indicating the maturation of fine motor control in human speech. It was thus proposed that, toddlers at the onset of speech development do not have fine control over rapidly changing speech sounds. A possible deviation from this typical developmental trajectory may be due to the presence of non-speech sounds in early life in children with autism, leading to an aberrant repertoire of sounds.

#### Number of vocalizations

In addition to the speech features, the speech samples from each toddler were used to calculate the number of vocalizations. Each vocalization was defined as a continuous string of speech sounds with no pause greater than 300 ms. For every toddler, this was evaluated by two listeners and the mean number of vocalizations for each toddler normalized with respect to duration of the sound file was used as a measure of number of vocalizations.

### Behavioral and diagnostic measures

#### Autism diagnostic interview-revised (ADI-R; Lord et al., 1994)

The ADI-R is a structured and standardized parent interview developed to assess the presence and severity of symptoms of autism in early childhood across all three main symptom domains: social relatedness, communication, and repetitive/restrictive behaviors. The ADI-R has been validated psychometrically across wide ranges of symptom severity.

#### Autism diagnostic observation schedule (ADOS; Lord et al., 1999)

The ADOS is a semi-structured, interactive schedule designed to assess social and communicative functioning among those who may have ASD. The schedule consists of four developmentally sequenced modules of which only one is administered, depending on the examinee's expressive language ability. Each module includes a standardized diagnostic algorithm composed of a subset of the social and communicative behavior, with lower scores indicating better functioning. Due to the age and language ability of the participants in the current study, all children were evaluated using either Module 1 or 2.

#### Mullen scales of early learning: AGS edition (Mullen, 1997)

The Mullen is a standardized measure for use with infants and preschool children from birth through age 68 months and assesses gross motor, visual reception, fine motor, receptive language, and expressive language abilities, yielding a composite score. For purposes of the current study, expressive and receptive language *T*-scores as well as fine motor *T*-scores were used. Additionally, a mean score from the language subscales was used as a measure of verbal IQ (VIQ) to further classify the ASD population into subgroups. The ASD group had significantly lower VIQ (*M* = 44, *SD* = 22) than the TD (*M* = 107, *SD* = 11) or the DD group (*M* = 73, *SD* = 13; *F* = 106.8, *p* < 0.001). Using the mean VIQ of the ASD group as a cut-off, the group was divided into high VIQ [with VIQ more than 44; HVIQ-ASD (*n* = 20)] and low VIQ [with VIQ less than 44; LVIQ-ASD (*n* = 19)] for all subsequent analyses.

#### Vineland adaptive behavior scales: survey form-expressive and receptive language subdomains (Sparrow et al., 1984)

The Vineland is a standardized parent interview that assesses adaptive behavior in four domains for children 6 years, 11 months of age and younger including communication skills, daily living skills, socialization, and motor skills. The Vineland was chosen as a measure of language in the current study based on previous research correlating it with other well-established measures of communication and language ability in young children (Stone et al., 1999; Rescorla and Alley, 2001; Toth et al., 2006). The subscale standard scores from the Expressive and Receptive Language subdomains were used.

### Statistics

One-Way ANOVAs were used to assess statistical differences among the three groups, ASD, TD, and DD, on the clinical and behavioral measures described in Table 1. To identify the effects of the different articulatory features, SR, FT, and POA, a single Kruskal–Wallis One-Way ANOVA collapsed across groups was performed. To explore group differences, One-Way ANOVAs were performed for each timescale: SR, FT, and POA. For the above ANOVA analysis, the ASD group was subdivided in HVIQ-ASD and LVIQ-ASD as described before, and for each of the timescales comparisons were made between HVIQ-ASD, TD, and DD and between LVIQ-ASD, TD, and DD independently. *Post-hoct*-tests with correction for multiple comparisons were performed to further explore effects of both group and timescale. Due to high variability in the toddler data, especially for the ASD group, descriptive statistics are provided to characterize the features of the POA distribution in the three groups (see Table 3). Additionally, in order to explore the relation between behavioral scores and articulatory features, Pearson's Correlation Coefficient was calculated for all three groups (Tables 2a,b,c). All analyses were performed in SPSS Version 20.0 (IBM, Corp., Armonk, NY) and SigmaStat 2.03 (Systat Software, San Jose, CA).

**Table 2:** **Correlations among language variables for children in the groups ASD, TD, and DD**.

**Articulatory feature**	**Mullen**			**Vineland**		**No. of vocalizations**
	**RL**	**EL**	**FM**	**RL**	**EL**	
**a. ASD GROUP**						
Syllabic rhythm	0.3	0.28	−0.45^*^	0.41^*^	0.24	−0.08
Formant transition	0.50^**^	0.29	−0.36	0.19	0.28	0.35^*^
Place of articulation	0.43^*^	0.03	−0.45^*^	0.27	0.1	0.08
**b. TD GROUP**						
Syllabic rhythm	0.57^**^	0.28	0.2	0.05	0.2	−0.47^*^
Formant transition	−0.06	−0.19	0.34	0.05	−0.05	−0.1
Place of articulation	0.03	0.1	0.3	0.16	0.26	0.02
**c. DD GROUP**						
Syllabic rhythm	−0.05	0.01	0.20	−0.14	−0.15	−0.004
Formant transition	−0.43	0.22	−0.10	−0.01	−0.01	−0.03
Place of articulation	−0.31	−0.13	−0.10	−0.16	−0.31	−0.02

Notes: Mullen RL, Mullen Scales of Early Learning Receptive Language T-Score; Mullen EL, Mullen Scales of Early Learning Expressive Language T-Score; Mullen FM, Mullen Scales of Early Learning Fine Motor T-Score; Vineland RL, Vineland Adaptive Behavior Scales Receptive Language Subscale Standard Score; Vineland EL, Vineland Adaptive Behavior Scales Expressive Language Subscale Standard Score;

* *p* < 0.05;

** *p* < 0.01.

## Results

A Kruskal–Wallis One-Way ANOVA for articulatory features at each timescale—SR, FT, and POA, collapsed across all participants, showed significant differences between timescales (*H* = 111.7, *df* = 2, *p* < 0.001). *Post-hoc* Tukey tests with correction for multiple comparisons showed significant differences between SR and FT (*p* < 0.05) and SR and POA (*p* < 0.05), but not between FT and POA, demonstrating that the contour area for SR was the highest in all participants. Kruskal-Wallis One-Way ANOVAs across groups (ASD, TD, and DD) for each of the three articulatory features, SR (*p* = 0.37), FT (*p* = 0.48), and POA (*p* = 0.22) did not show any significant effects of group. This was possibly because of the high variability in the ASD data, which led to loss of statistical power. Due to the high variability in the ASD group, we subdivided them into LVIQ-ASD and HVIQ-ASD based on a measure of verbal ability. On performing a One-Way ANOVA between the LVIQ-ASD, TD, and DD groups for each of the articulatory features, we found that there was a significant effect of group (*F* = 3.98, *df* = 2, *p* = 0.029) for the shortest-timescale measure, POA. *Post-hoc* comparisons using *t*-tests with corrections for multiple comparisons using Fisher LSD method showed differences between LVIQ-ASD and TD (*p* = 0.03) as well as LVIQ-ASD and DD (*p* = 0.02), with the LVIQ-ASD group having the largest area for POA. There were no differences between DD and TD (*p* = 0.78) groups. However, when we compared the HVIQ-ASD, DD, and TD groups for the same variable, we found no significant differences (*p* = 0.86). To further explore the variability in all three groups, descriptive statistics were computed for the shortest-timescale measure, POA, which showed the highest variability and was of interest from a developmental perspective. The characterization of data in the three groups for all three features is shown in Table 3. The variability of the ASD group was the highest as compared to TD and DD as measured by the standard deviation, confidence interval of the mean and the range of the POA data. From a previous study (Singh and Singh, 2008), it emerged that in the course of TD there is very little power in the rapid timescale features like POA even at 4 years of age. Our results showed that for all three groups, the long-timescale feature, SR (2–10 Hz), had the largest area enclosed with no significant differences across the three groups. There were also no significant differences across the three groups for FT (25–40 Hz). However, for the shortest-timescale feature, POA (50–100 Hz), the ASD group exhibited larger areas enclosed in comparison to both the TD and DD groups (see Figure 3D). Our findings show that a subgroup of the ASD population, who have poor verbal skills had significantly larger areas for the shortest-timescale feature demonstrating that this change in POA is significantly related to a measure of language skills. We propose the hypothesis that this deviance in the ASD articulatory features maybe due to the presence of aberrant or non-speech sounds in their vocalizations (Wolk and Giesen, 2000) and is possibly reflected in atypical power in the rapidly changing timescales, a feature that is absent in typical toddlers.

**Table 3:** **Descriptive statistics for the place of articulation contour areas of ASD, TD, and DD group**.

**Statistic**	**ASD (***n*** = 39**)	**TD (***n*** = 26**)	**DD (***n*** = 20**)
Mean	60.7	37.8	22
Standard deviation	101.6	57.6	36.4
Standard error of mean	16.3	11.3	8.1
C.I. of mean	32.9	23.3	17.1
Range	407	106	206
Normal distribution	No	No	No

**Figure 4:**
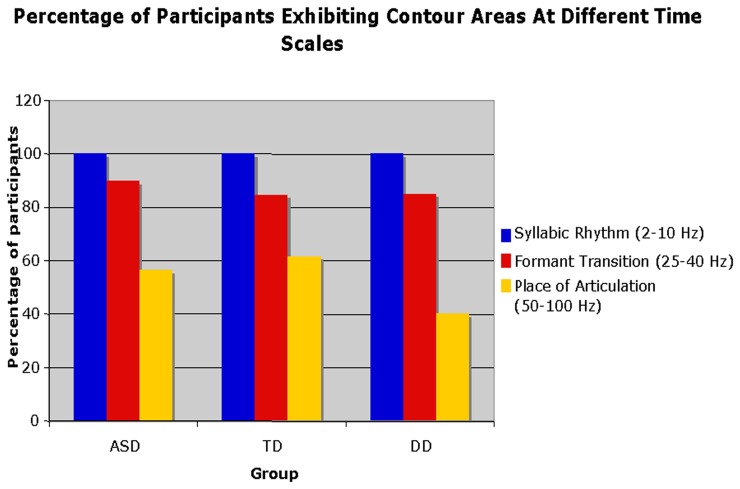
**The percentage of participants of each of the three groups (ASD, typical development, developmental delay) exhibiting contour areas at different time scales**.

An additional finding indicated that across all three groups, the percentage of participants exhibiting power for an articulatory feature decreased as the feature became shorter in timescale (see Figure 4). For example, while 100% of participants in each of the three groups exhibited power in the longest-timescale feature (SR), for shorter-timescale features, such as FT and POA, the general trend was a decrease in the percentage of participants exhibiting power for those features. The decrease in power exhibited for rapidly changing spectro-temporal modulations may reflect the level of maturity of speech-motor skills and changes with age in the TD group. This is consistent with previous findings for typically developing children indicating that the appearance of such features are age-dependent, and that adult-like speech-motor patterns do not appear until ~6–7 years of age (Singh and Singh, 2008). However, there are qualitative differences in the power exhibited by typically developing children with mature speech motor skills and the increase in power exhibited by our ASD toddler cohort. Specifically, these differences lie in the shape of the contour enclosed by vocalizations of the toddlers from different groups. The TD group show typical, matured contours exhibiting energy in regions along the axes which encode “speech sounds,” whereas the regions of the speech modulation spectrum space occupied by the ASD groups are spread within the quadrant and encode more “non-speech” and “noise-like” information (Singh and Theunissen, 2003; Singh and Singh, 2008). A detailed analysis of these differences is beyond the scope of this article. Although participants across groups exhibited similar trends in the presence of the three articulatory features discussed above, the contour areas of each feature at different timescales differed among groups, although not significantly. Children with ASD showed an atypical pattern of articulatory feature development and exhibited greater contour areas in features associated with shorter-timescales than the TD and DD groups.

### Number of vocalizations

The number of vocalizations elicited by toddlers in each group was compared. A One-Way ANOVA showed significant differences across the three groups (*F* = 13.21, *df* = 2, *p* < 0.001). *Post-hoc* Tukey tests showed significant differences between number of vocalizations for ASD and TD, and DD and TD (*p* < 0.05), with the ASD group eliciting the fewest number of vocalizations and the TD group the highest. There were no significant differences between the ASD and DD groups.

### Correlational analyses

Groups significantly differed from each other in terms of their language ability as measured by the Mullen and the Vineland. Correlations between contour areas for the three articulatory features, number of vocalizations, and all standard measures of language ability were examined for all three groups (see Tables 2a,b,c). For the ASD group, receptive language ability, as measured by the Mullen Scales, was significantly correlated with total contour area, FT, and POA. In addition, there was a significant correlation between SR and both the Vineland Receptive Language subscale and the Fine Motor scale of the Mullen Scales as well as between FT and number of vocalizations. Additionally, the POA in ASD also correlated with Fine Motor scale on the Mullen Scales. For the TD group, the only significant correlation was found between receptive language ability, as measured by the Mullen Scales, and SR. No significant correlations between contour areas and measures of language ability were found for the DD group.

## Discussion

In the current study, a free play scenario was used to collect naturalistic speech samples for toddlers with ASD, DD, and TD from which measures of speech motor function were obtained. Using spectral analysis, speech samples from all participants were examined for different articulatory features, which carry information about speech motor abilities at different timescales. Our findings showed that all our participants, namely, toddlers with ASD, typically developing toddlers, as well as those with DD, exhibited a decrease in contour area with increasing timescale of spectro-temporal modulation change. Participants also showed similar spectro-temporal distributions for the long-timescale articulatory features such as SR (2–10 Hz) as well as FT (Figure 3). However, group differences were observed for shortest-timescale feature (50–100 Hz) reflective of POA in a subgroup of ASD toddlers who had significantly poorer language skills. In a previous study, the refinement of fine motor control of speech was reflected in the presence of power in this shorter-timescale feature of POA. However, the shape of the contour in the ASD group, reflecting power in POA is significantly different and may reflect a function other that just maturational control of speech. For instance, the presence of atypical blends and differently uttered sounds in the ASD speech repertoire, maybe additionally be causing these differences. Furthermore, the heterogeneity of the ASD sample is reflected in the high variability and non-Gaussian nature of the distribution (Table 3). This variability could be explored further in the context of varying levels of receptive and expressive language ability in the ASD population, as demonstrated by our subgroup analysis. Our results are consistent with recent findings demonstrating no differences in the syllabic structure complexity produced by typically developing children and those with ASD (Schoen et al., 2011), but significantly fewer consonant blends, greater number of atypical blends in ASD speech (Schoen et al., 2011), and differences in the nature of uttered syllables (Shriberg et al., 2011). If such atypical features can be identified in children with ASD during the toddler period, it may be possible to use this measure not only as an early risk indicator of ASD, but also to predict the developmental trajectory of speech motor development and individual responses to language-related intervention.

Another noteworthy point is the substantial heterogeneity in the articulatory features demonstrated by the ASD group. It is well-known that ASD is extremely heterogeneous in its presentation with significant variability in the area of language abilities. While some individuals with ASD are verbally fluent and meet their language developmental milestones on time, 30–50% of children with ASD are reported to have significant impairments in language and/or remain non-verbal into adulthood (Howlin et al., 2004). However, additional research suggests that the proportion of non-verbal children with ASD is less than 20% for those children who are referred for evaluation of ASD at early ages (Lord et al., 2004), illustrating the importance of early detection and diagnosis. As illustrated by our results, the analysis of the LVIQ and HVIQ subgroups of ASD further confirms the variability in the ASD population and demonstrates the need to identify subgroups with specific defining characteristics within the autism spectrum to develop more sensitive and specific measures of early diagnosis and identification.

Within the ASD group, correlations between contour areas for the three articulatory features and measures of language ability revealed an interesting pattern of results. The longest-timescale feature, SR, was significantly correlated with both receptive language ability, as measured by the Vineland, and fine motor skills, as measured by the Mullen. The shorter-timescale features, FT and POA, were both significantly correlated to receptive language ability as measured by the Mullen. In addition, the POA measure was also significantly correlated with fine motor skills as evaluated on the Mullen. Given that that there were significant differences in the POA feature in the ASD group as compared to DD and TD, this finding may be significant in understanding the role of motor development in speech output during development.

When interpreting the results of the correlation analysis, it is important to note characteristics of the participants in the current sample, including their chronological age, VIQ, ASD diagnosis, and associated communication deficits. For example, the Vineland and Mullen receptive language subscales for toddler-aged children evaluate a child's ability to orient or attend to verbal and social stimuli, their understanding of simple words and instructions (i.e., “no,” “yes,” names of familiar people, “where's the door?”), their use of gestures in response to simple commands (i.e., raising their arms when a caregiver says “Come here” or “Up”), and the presence of echolalia or atypical prosody. Many of these receptive and non-verbal language skills are fundamental building blocks for expressive language development and are often delayed in children with ASD (Tager-Flusberg, 1996; Howlin, 2003; Tager-Flusberg and Joseph, 2003; Eigsti et al., 2007). In the area of receptive language, retrospective parent reports indicate that children with ASD understood fewer phrases than developmentally delayed or typically developing children by age 24 months (Luyster et al., 2008). Prospective studies indicate similar impairments in early language comprehension. For example, high-risk infant siblings later diagnosed with ASD showed decreased vocabulary comprehension and fewer phrases understood as measured by the McArthur Communicative Development Inventories (MCDI; Fenson et al., 1993) between 12 and 24 months of age (Mitchell et al., 2006; Stone et al., 2007). The presence of significant delays in language comprehension, therefore, has implications for concomitant as well as future adaptive functioning and non-verbal social communication skills (Rutter et al., 1992; Tager-Flusberg et al., 2005).

Language deficits characteristic of ASD, as described above, were demonstrated in the current study. For measures of both receptive and expressive language on the Mullen Scales and Vineland, our findings revealed significant differences between ASD, TD, and DD groups, with children with ASD demonstrating the most severe impairments. It is important to note that despite these differing levels of language ability, the speech articulatory features measure used in this study is designed to capture the qualitative differences for any speech sounds (including both vocalizations and attempted or actual word use). Therefore, the significant correlations found between speech features and receptive language ability for the ASD group suggests a unique marker for this group rather than a result of the ASD children simply having more extensively delayed language development. However, we do recognize the need for future studies to examine speech features in 3–5 year old children with ASD in order to substantiate associations between speech features and language ability in this population as expressive language develops. Furthermore, longitudinal studies may be useful in exploring the developmental trajectory between speech features and receptive and expressive language abilities (i.e., “Do correlations between speech features and receptive language abilities predict future delays in expressive language or correlations between expressive language and speech features?”).

Current research on toddler vocalizations mainly uses transcription, which is a laborious and time consuming process and subject to variability. One of the objectives of this study was to use a semi-automated algorithm for labeling vocalizations using the timescale of spectro-temporal change as a parameter, in order to simplify the process of speech analysis and reduce its subjectivity. Future work correlating data from this method with existing transcription codes will further validate the use of this method.

Our findings add to previous research on speech motor function by examining these features in a sample of toddlers that included typically developing children, children with DD without ASD, and children with ASD. Speech features were compared among these groups, revealing significant differences for the shorter-timescale feature of POA for the ASD group as compared to both the TD and DD groups. Overall, results suggest that toddlers with ASD show abnormal patterns in articulatory features as compared to both typically developing and developmentally delayed children. Additionally, significant concurrent correlations were found between both longer- and shorter-timescale articulatory features and receptive language domains on the Mullen and Vineland. Although our findings suggest the use of a novel method of assessing speech motor development in children as an early screening measure, there are some limitations of the method in its current form. Future research demonstrating replicability and reliability of the method in different samples is needed to establish speech features as an additional, useful measure of individual differences in vocalization patterns among children with ASD.

## Conflict of interest statement

The authors declare that the research was conducted in the absence of any commercial or financial relationships that could be construed as a potential conflict of interest.
